# *Corynebacterium matruchotii* Demography and Adhesion Determinants in the Oral Cavity of Healthy Individuals

**DOI:** 10.3390/microorganisms8111780

**Published:** 2020-11-13

**Authors:** Anders Esberg, Angela Barone, Linda Eriksson, Pernilla Lif Holgerson, Susann Teneberg, Ingegerd Johansson

**Affiliations:** 1 Department of Odontology/Section of Cariology, Umeå University, SE 901 87 Umeå, Sweden; linda.eriksson@umu.se (L.E.); ingegerd.johansson@umu.se (I.J.); 2 Institute of Biomedicine, Department of Medical Biochemistry and Cell Biology, Sahlgrenska Academy, University of Gothenburg, SE 405 30 Göteborg, Sweden; angela.barone@gu.se (A.B.); susann.teneberg@medkem.gu.se (S.T.); 3 Department of Odontology/Section of Pedodontics, Umeå University, SE 901 87 Umeå, Sweden; pernilla.lif@umu.se

**Keywords:** *Corynebacterium matruchotii*, demographics, aggregation, ligand, glycolipids

## Abstract

*Corynebacterium matruchotii* may be key in tooth biofilm formation, but information about demographics, bacterial partners, and binding ligands is limited. The aims of this study were to explore *C. matruchotii*’s demography by age and colonization site (plaque and saliva), in vitro bacterial–bacterial interactions in coaggregation and coadhesion assays, and glycolipids as potential binding ligands in thin-layer chromatogram binding assays. *C. matruchotii* prevalence increased from 3 months to 18 years old, with 90% and 100% prevalence in saliva and tooth biofilm, respectively. *C. matruchotii* aggregated in saliva in a dose-dependent manner but lacked the ability to bind to saliva-coated hydroxyapatite. In vivo, *C. matruchotii* abundance paralleled that of *Actinomyces naeslundii, Capnocytophaga* sp. *HMT 326*, *Fusobacterium nucleatum* subsp. *polymorphum*, and *Tannerella* sp. *HMT 286.* In vitro, *C. matruchotii* bound both planktonic and surface-bound *A. naeslundii*, *Actinomyces odontolyticus*, and *F. nucleatum*. In addition, *C. matruchotii* exhibited the ability to bind glycolipids isolated from human erythrocytes (blood group O), human granulocytes, rabbit intestine, human meconium, and rat intestine. Binding assays identified candidate carbohydrate ligands as isoglobotriaosylceramide, Galα3-isoglobotriaosylceramide, lactotriaosylceramide, lactotetraosylceramide, neolactotetraosylceramide, and neolactohexaosylceramide. Thus, *C. matruchotii* likely uses specific plaque bacteria to adhere to the biofilm and may interact with human tissues through carbohydrate interactions.

## 1. Introduction

*Corynebacterium matruchotii* (previously *Bacterionema matruchotii)* [1] is a Gram-positive actinobacterium with long filaments and a typical “wipe-handle” morphology [1]. *C. matruchotii* has previously been found in a morphological unit in tooth biofilms, termed “corn cobs”, formed by the filamentous organism with adherent cocci [2]. Recent studies redefined the bacterial organization in the supragingival biofilm as a hedgehog-like structure and suggested that *C. matruchotii* is a nucleating species in the bacterial community [3]. Morphological characterization of the hedgehog structure has revealed a network in which *Streptococcus* and *Actinomyces* species attach to the *C. matruchotii* filaments, with more cocci adhering to its distal filament tips [3]. Other species in the hedgehog structure belong to *Porphyromonas*, *Haemophilus, Aggregatibacter,* *Neisseria*, *Fusobacterium,* and *Leptotrichia* genera [3]. However, Kolenbrander and Williams [4] did not observe any interaction between *C. matruchotii* and *Actinomyces viscosus* (strains MG1, T14V, and T14AV), *Actinomyces naeslundii* (strains ATCC 12104, I, and W1544), or *Streptococcus* spp. (strains DL1, Challis, Hi, 34, and J22). Kim and Koo [5] recently confirmed that the corncob, hedgehog, and seaweed spatial architectures are common, but also documented a dome-like structure in caries-active toddlers [6].

Several 16S rRNA gene sequencing studies have found *C. matruchotii* among the most prevalent species in the adult human oral core microbiome, as they are present in virtually all subjects in significant abundance [7,8]. This was also confirmed in metaproteome and metatranscriptome studies [9,10]. Furthermore, *C. matruchotii* represents a large proportion of the protein activity in human tooth biofilms [9,10] and secretes a 50 amino acid membrane-associated proteolipid inducing calcium precipitation [11], as well as an oxidoreductase (MdbA) that catalyzes a disulfide bond formation in vitro, which may be utilized to catalyze oxidative protein folding and stabilization [12].

The relationship between *C. matruchotii* and oral health remains unclear. Some studies report *C. matruchotii* to be enumerated in caries-free children and young adults [7,13], and reduced in caries progression [13]. However, a yet unnamed *Corynebacterium* phylotype has been found to be among the predominant species related to caries progression in the primary and permanent dentition [14]. Similarly, some studies have found an association between *C. matruchotii* and periodontal health [15,16], but one study reported that *C. matruchotii* is more than twice as prevalent in smokers, indicating an association or co-appearance with periodontal disease, which is more common in smokers [17].

Thus, support is growing for a possible key role of *C. matruchotii* in oral biofilm formation, but much remains to be understood about its role in oral microbiota biology and oral health. The aims of this study were to explore *C. matruchotii*’s demography by age and colonization site (plaque and saliva), in vitro bacterial–bacterial interactions in coaggregation and coadhesion assays, and glycolipids as potential binding ligands in thin-layer chromatogram binding assays.

## 2. Materials and Methods

### 2.1. Study Population

Data on the prevalence and abundance of *C. matruchotii* and other oral bacteria were extracted from previous study cohorts: One study on age transformation of the oral microbiota [18] and one employing young adults [19]. The cohorts were described in their respective publications. Both studies with addendums were approved by the Swedish Ethical Review Authority, Sweden (Dnr 2011-90-31 M, 2012-111-31 M, 2015-389-32 M, 2017-450-31, and 2016-239-32 M) and followed the Declaration of Helsinki and the General Data Protection Regulation (GDPR). All parents and young adults were given written and verbal information before signing informed consent to participate and agreeing to the use of the information in research.

### 2.2. Biological Samples and Information on Medical, Lifestyle, and Living Conditions

Saliva swabs, whole chewing stimulated and parotid saliva, tooth biofilm, breast milk, and feces were sampled for microbiota characterization as described elsewhere [18,20]. Briefly, saliva swab samples were collected by swirling sterile cotton swabs in the mouth of 2-day, 3-month, 18-month, 3-year, and 5-year-old children, followed by dispersal of bacteria in 10 mM Tris-HCl, 1 mM disodium EDTA, pH 7.4. Milk was collected by the mothers after carefully cleaning the nipple with soap and water and drying with a clean towel. Feces from the children was sampled by the parents at home using tubes designed for this purpose (NC9574115, Sarstedt, Inc. SC TUBE CBS, Fisher Scientific, Gothenburg, Sweden). Written and verbal information were given to the mothers prior to sampling. Milk and feces samples were stored at −20 °C for approximately 2 weeks and then transported on ice to the lab and transferred to a −80 °C freezer until prepared for analysis.

From the young adults (18–23 years old), supragingival biofilm was collected from accessible tooth surfaces using sterile wooden toothpicks. Tooth biofilm samples were pooled by subject in buffer and the participants abstained from oral hygiene on the morning of the sampling. Whole chewing stimulated saliva was collected for 3 min in sterile test tubes.

All samples were immediately transferred to −80 °C freezers.

Information on medical and other conditions was collected via questionnaires. The young adults also reported their habitual dietary intake on a 93-question semi-quantitative food frequency questionnaire (FFQ, http://www.matval.se) as described previously [20]. Intake of energy and energy-providing nutrients were estimated as described previously [21].

For in vitro experiments, parotid saliva was collected from volunteers in ice-chilled test tubes using Lashley cups and with citric acid stimulation.

### 2.3. DNA Extraction and Microbiota Sequencing

Genomic DNA extraction from saliva swabs, saliva, tooth biofilm, milk, and feces samples and positive controls, PCR amplicon and library construction, Illumina sequencing, and sequence accession numbers are described in Appendix A and in the basic references [18,20].

### 2.4. Bacterial Cultures

*C. matruchotii* strains (CCUG46620 and CCUG47160) were grown on sheep blood agar plates for 48 h, washed once with 10 mM Tris-base-saline buffer (TBS) pH 7.6, and adjusted to an optical density of 1.0 at 600 nm (OD_600_, ~10^9^ colony forming units (CFU)/mL) for use in coaggregation assays. For ^35^S-labeling, *C. matruchotii* was grown on blood agar plates supplemented with ^35^S-methionine/cysteine (NEG709A, PerkinElmer, Hägersten, Sweden), washed twice, and adjusted to an optical density of 0.2 at OD_600_ before use. For chromatogram binding assays, the bacteria were cultured on blood agar plates and radiolabeled by the addition of 50 μCi ^35^S-methionine (PerkinElmer; NEG77207MC) diluted in 0.5 mL phosphate-buffered-saline (PBS) pH 7.3 to the culture plates. After incubation for 12 h at 37 °C, the bacteria were harvested, centrifuged three times with PBS, and then suspended in PBS containing 2% (*w/v*) bovine serum albumin, 0.1% (*w/v*) NaN_3_, and 0.1% (*w/v*) Tween 20 (BSA/PBS/TWEEN) to a bacterial density of 1x108 CFU/mL. The specific activity of the suspensions was approximately 1 cpm per 100 bacteria.

For fluorescein isothiocyanate (FITC) labeling, *C. matruchotii* was grown overnight on blood agar plates; washed once in PBS, supplemented with 0.1% Tween-20 (PBS-T); resuspended in 0.1 M carbonate 0.15 M NaCl_2_ buffer to an optical density of 1.0 at OD_600_; incubated with 10 mg/mL FITC (F7250, Sigma–Aldrich, Stockholm, Sweden) for 8 min, followed by three washes in PBS-T; and finally adjusted to 1.0 at OD_600_. Tested bacterial partner strains were grown on blood, Rogosa, or chocolate agar plates under aerobic or anaerobic conditions (Table S1). Bacteria were harvested after 48 h of growth, washed once with TBS, adjusted to an optical density of 0.5 at OD_600_, yielding approximately 10^8^ CFU/mL, and frozen at −80 °C until use.

### 2.5. Fluid Phase Coaggregation

Saliva-mediated aggregation and bacteria–bacteria coaggregation were tested in a sedimentation assay in which equal volumes (0.1 mL) of *C. matruchotii* and parotid saliva or test strains were mixed in plastic round-bottom 2 mL Eppendorf tubes. The tubes were vortexed for 15 s and incubated for 10 min to allow coaggregates to form. The aggregates were scored qualitatively (++ for large, + medium, (+) small, and – no aggregates). The sedimentation assay was also used to quantify saliva-mediated aggregation by following OD_600_ over time in a spectrophotometer after mixing equal volumes of saliva (0.5 mL of varying concentration) and *C. matruchotii* (OD_600_ = 2.0).

### 2.6. Surface-Based Binding

Nitrocellulose membranes (18Ω, GE Health Care, Hybond ECL, RPN303D) were pre-wetted for 1 min, equilibrated in TBS for 10 min, and mounted in a microfiltration dot blot device (Bio-Dot Apparatus, 1706545, BioRad, Solna, Sweden). Serial dilutions of bovine serum albumin (BSA, Sigma A2153), parotid saliva, and breast milk (1:2 to 1:256 in TBS), suspensions of potential partner bacteria (50 μL, OD_600_ 0.1 in TBS diluted from 0.1 to 0.004 in TBS, Table S1), and negative controls (TBS) were applied to assigned wells and mounted on the membrane using vacuum suction. The membranes were removed from the slot blot, rinsed, and washed (TBS, 0.1% Tween 20 (TBS-T)) before being blocked (ECL Prime blocking agent, RPN418V, GE Health Care) for 1 h. The membranes were rinsed briefly before FITC-labeled *C. matruchotii* was applied (50 mL, OD_600_ = 1.0) and allowed to bind to potential partners for 2 h at 4 °C in the dark. Finally, the membranes were rinsed and washed (3 × 5 min) in TBS-T and fluorescent signals detected using the ChemiDoc MP Imaging system (Bio-Rad).

### 2.7. Hydroxyapatite Adhesion and Coadhesion Assay

Adhesion of metabolically ^35^S-labeled *C. matruchotii* to hydroxyapatite beads (Bio-Rad) coated with parotid saliva, breast milk, or saliva-partner bacteria was measured as described previously [22]. The assay served as a tooth tissue model. Briefly, hydroxyapatite beads hydrated overnight (5 mg beads in 125 μL adhesion buffer (1 mM KH_2_PO_4_-K_2_HPO_4_ buffer, pH 6.5, containing 50 mM KCl, 1 mM CaCl_2_, and 0.1 mM MgCl_2_) per 96 wells) were coated with 125 μL parotid saliva or breast milk for 1 h, washed twice in adhesion buffer, and blocked with 5% BSA in adhesion buffer. Parotid saliva was pooled from up to 10 donors and serial dilutions in adhesion buffer from 1:2 to 1:256 were tested. The blocked coated beads were incubated with ^35^S-labeled *C. matruchotii* (125 μL, 10^8^ cells/mL) for 1 h at room temperature under agitation, washed three times, resuspended in scintillation solution, and counted using MicroBeta^2^ (PerkinElmer, Hägersten, Sweden). The proportion of bound bacteria out of the total amount of added bacteria (percent adhesion) was calculated.

To evaluate whether bacterial partners could recruit *C. matruchotii* to saliva-coated hydroxyapatite surfaces, an unlabeled co-bacterial partner was bound to BSA-blocked saliva-coated hydroxyapatite beads, washed three times in adhesion buffer, and subsequently incubated for 1 h with metabolically ^35^S-labeled *C. matruchotii* (125 μL, 10^8^ cells/mL). Finally, the beads were washed three times in adhesion buffer, resuspended in scintillation solution, and counted using MicroBeta^2^ (PerkinElmer). The proportion of bound bacteria out of the total amount of added bacteria (%-adhesion) was calculated and compared to *C. matruchotii* binding to saliva-coated beads.

### 2.8. Chromatogram Binding Assays

Reference glycolipids were isolated as described previously [23] and characterized by mass spectrometry [24,25] and ^1^H-NMR spectroscopy [26]. Thin-layer chromatography was performed on aluminum-backed silica gel 60 high performance thin-layer chromatography plates (Merck, Stockholm, Sweden). Glycosphingolipid mixtures (10–40 μg) or pure glycosphingolipids (4 μg) were applied to the plates and eluted with chloroform/methanol/water (60:35:8, by volume). Chemical detection was achieved with anisaldehyde [27].

Radiolabeled bacteria were bound to glycosphingolipids on thin-layer chromatograms as described previously [28]. Dried chromatograms were dipped in diethyl ether/*n*-hexane (1:5 *v/v*) containing 0.5% (*w/v*) poly(isobutyl methacrylate) for 1 min. The chromatograms were blocked with BSA/PBS/TWEEN for 2 h at room temperature before incubating the plates for 2 h at room temperature with ^35^S-labeled bacteria (1–5 × 10^6^ cpm/mL) diluted in BSA/PBS/TWEEN. After washing six times with PBS and drying, the plates were autoradiographed for 12–36 h using XAR-5 x-ray films (Carestream; 8941114).

### 2.9. Statistical Analysis

Quantitative variables were presented as means; median with standard deviations and group differences tested by Student’s *t*-test. Spearman correlation coefficients were used to assess co-variations between *C. matruchotii* and bacteria in saliva or tooth biofilms. These analyses were performed using SPSS version 26 (IBM Corporation, Armonk, NY, USA). The tests were two-tailed and *p*-values < 0.05 considered significant. Taxa associated with high levels of *C. matruchotii* were identified in multivariate partial least squares (PLS) regression. The results are presented in a PLS loading plot with influential variables (i.e., a variable importance in projection (VIP) score >2.0). The PLS models evaluated the abundance of *C. matruchotii* in saliva and tooth biofilm as dependent variables (y-variable) against an independent x-block composed of all other identified bacterial species (x-variables, *n* = 311). All variables utilized for PLS regression analysis were auto-scaled and logarithmically transformed as needed to improve normality. SIMCA P+ version 15.0 (Umetrics AB) was used for these analyses.

## 3. Results

### 3.1. C. matruchotii Demography by Age and Sample Type

**Figure 1:**
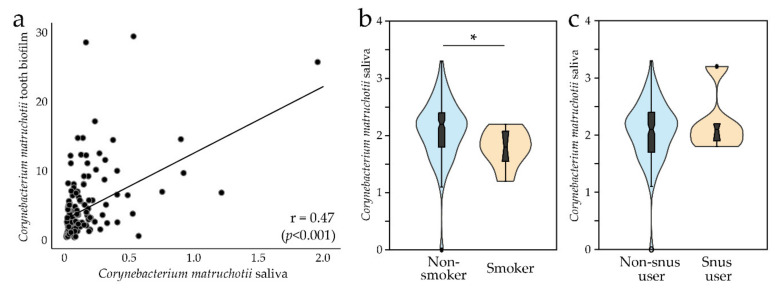
(**a**) Scatter plot of *C. matruchotii* abundance in saliva versus tooth biofilm. (**b**) Violin plots with a box plot comparing *C. matruchotii* abundance in saliva by smoking and (**c**) use of Swedish snus (snuff). * *p* < 0.05.

Detection of *C. matruchotii* in saliva was surveyed from early infancy to early adulthood (Table 1). All included participants were healthy, none had taken any antibiotic for at least 3 months before sampling, and no child was pre-term (birth weight > 2500 g) [18,20]. At 2 days and 3 months of age, *C. matruchotii* was virtually absent in saliva swabs (Table 1). At 18 months of age, *C. matruchotii* was detected in 50% of the children; thereafter, the detection prevalence increased up to 67% at 5 years of age. At 18 years of age, 98% and 100% had *C. matruchotii* in saliva and tooth biofilm samples, respectively, making *C. matruchotii* part of both the saliva and tooth biofilm core microbiome at least from 18 years of age. Though saliva detection prevalence of *C. matruchotii* varied by age, mean abundance was similar in carriers regardless of age (Table 1). When the infants were 3 months old, 10% of the mothers had *C. matruchotii* in their breast milk (Table 1). *C. matruchotii* was not detected in feces from 3-month or 5-year-old children (Table 1). Strikingly, the *C. matruchotii* abundance was 16 times higher in tooth biofilms than in saliva (i.e., 5.2% compared to 0.32%). The Spearman correlation coefficient between saliva and tooth biofilm abundance was 0.47 (*p* < 0.001, Figure 1a), and the linear regression coefficient with tooth biofilm abundance as the dependent and saliva abundance as the independent variable was 3.0 (95% CI 2.1, 3.8; *p* < 0.001).

### 3.2. C. matruchotii and Lifestyle Factors

We found no association between *C. matruchotii* abundance and early infancy feeding mode (breast feeding or not) or birth mode (vaginally or by caesarian section), use of pacifier, or the way the parents cleaned the pacifier (all evaluated at 18 months of age) (Table S2). Young adults who smoked had significantly lower abundance of *C. matruchotii* in saliva (mean abundance (log value) 1.77 versus 2.09, median 1.79 versus 2.16, *p* = 0.032, Figure 1b). The levels did not differ between those who used Swedish snus (snuff) and those who did not (Figure 1c). Differences by tobacco use could not be tested for tooth biofilm samples because too few participants with tooth biofilm samples used tobacco. Furthermore, no association was found between *C. matruchotii* abundance and number of teeth, saliva flow rate, caries scores, or intake of sugar or any other food or dietary component in the young adults (Table S2).

### 3.3. Saliva and Breast Milk Mediated Fluid Phase Aggregation and Solid Phase Adhesion of C. matruchotii

**Figure 2:**
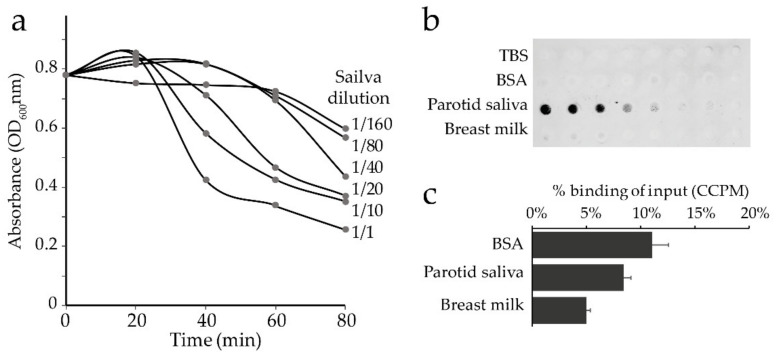
Saliva and breast milk aggregation and adhesion of *C. matruchotii*. (**a**) Sedimentation assay of aggregation in parotid saliva by time and (**b**) binding of Tris-base-saline buffer (TBS) (Ctrl), bovine serum albumin (BSA, 2.5 mg to 0.020 mg per mL TBS), pooled parotid saliva (diluted 1:2, 1:4, 1:8, 1:16, 1:32, 1:64, 1:128, 1:256 in TBS), and pooled fat-free breast milk (diluted 1:2, 1:4, 1:8, 1:16, 1:32, 1:64, 1:128, 1:256 in TBS). (**c**) Proportion (%) S^35^-labeled *C. matruchotii* bound to BSA-, saliva-, or breast milk-coated hydroxyapatite beads. CCPM; Corrected counts per minute.

*C. matruchotii* (CCUG46620) aggregation in parotid saliva was evaluated using a sedimentation assay, and saliva- or breast milk-mediated adhesion was evaluated by a surface overlay assay and by binding to saliva- or breast milk-coated hydroxyapatite. Saliva aggregated *C. matruchotii* in a dose-dependent manner (Figure 2a). *C. matruchotii* also bound to saliva-coated nitrocellulose membranes (Figure 2b) but had a limited ability to bind to saliva coated on hydroxyapatite (Figure 2c). Breast milk did not mediate binding of *C. matruchotii* when coated on nitrocellulose membranes (Figure 2b) or hydroxyapatite beads (Figure 2c).

### 3.4. In Vivo Associations between C. matruchotii and Other Oral Bacteria

**Figure 3:**
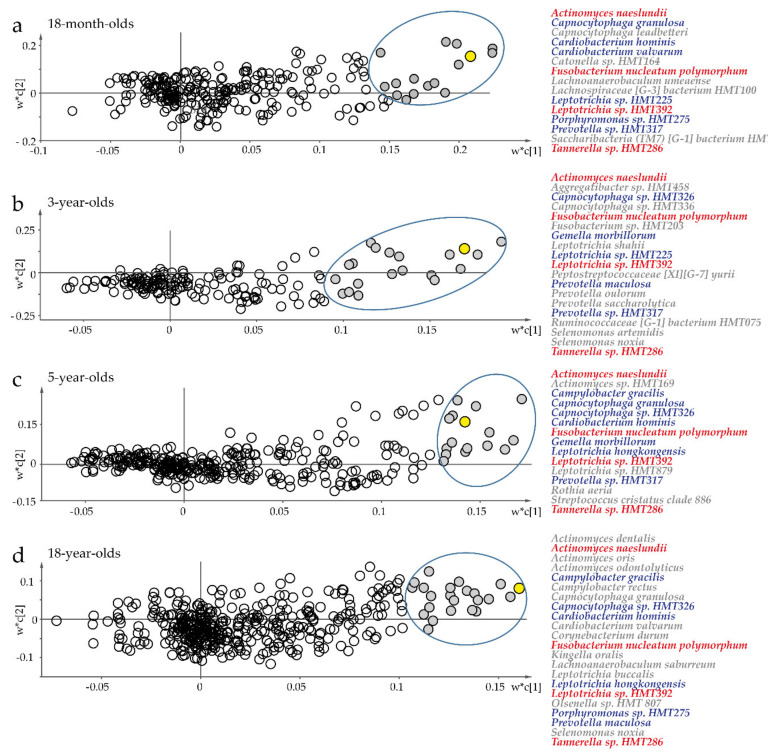
Multivariate partial least squares (PLS) regression of saliva microbiota to target potential partner bacteria for *C. matruchotii*. Scatter plots of PLS loading based on a model with *C. matruchotii* abundance as the dependent variable (Y, yellow filled symbol) and abundance of all other detected species in saliva swab samples as the independent variable block (X) for (**a**) 18-month-old infants, (**b**) 3-year-old children, (**c**) 5-year-old children, and (**d**) young adults. Unfilled symbols represent species with variable importance in projection (VIP) <2.0. Red text indicates species that were found in saliva at all ages, and blue indicates those found at two or three ages. w* describes the PLS weights from the combination of the original variables in the X-swarm; *c* is the component number. Species with a significant VIP score >2.0 are indicated.

To identify potential biofilm bacteria partners for *C. matruchotii* in biofilm formation, multivariate PLS modeling was performed by age strata with *C. matruchotii* abundance as the dependent variable. All models were very strong, with an explanatory power (R^2^) > 74% and predicted power (Q^2^) > 53%. Four bacterial species were strongly associated (significant VIP values > 2.0) with saliva abundance of *C. matruchotii* at all ages (Figure 3a–d; Table S3): *A. naeslundii, F. nucleatum* subsp. *polymorphum, Leptotrichia* sp. *HMT 392,* and *Tannerella* sp. *HMT 286.* However, single species were similarly strongly associated with *C. matruchotii* abundance at two or three separate ages (Figure 3a–d; Table S3). Similar to saliva, very strong associations (VIP > 2.0) were seen between *C. matruchotii* abundance and *A. naeslundii* and *Tannerella* sp. *HMT 286* in tooth biofilm samples from young adults, and with *Actinomyces massiliensis, Actinomyces odontolyticus* (sp. *HMT 178*), *Actinomyces* sp. *HMT 171, Campylobacter gracilis Capnocytophaga* sp. *HMT 336, Cardiobacterium hominis, Cardiobacterium valvarum, Lachnoanaerobaculum saburreum*, *Leptotrichia hofstadii, Leptotrichia wadei,* and *Selenomonas noxia* in saliva samples (Table S3). Univariate Spearman correlation analyses confirmed the associations identified by PLS in saliva and tooth biofilm.

### 3.5. Fluid Phase C. matruchotii Coaggregation with Other Bacterial Species

*C. matruchotii* was present in all tooth biofilm samples but did not present strong binding to saliva-coated hydroxyapatite; therefore, we hypothesized that *C. matruchotii* utilizes one or several partner bacteria to manifest in the tooth biofilm. A set of species were selected from the in vivo multivariate PLS screening (Figure 3) and previously reported interactions with *C. matruchotii* [3]. The first step was to test a panel of potential candidate species (83 strains in 47 species) for coaggregation with planktonic *C. matruchotii* (Table S4).

Coaggregation occurred between *C. matruchotii* and strains of *A. naeslundii*, *Actinomyces odontolyticus*, *Corynebacterium durum*, *Fusobacterium nucleatum* subsp. *nucleatum*, *Fusobacterium nucleatum* subsp. *polymorphum*, *Fusobacterium periodonticum*, and *Porphyromonas gingivalis*; weak coaggregation was seen for *Aggregatibacter actinomycetemcomitans*, *Gemella haemolysans*, *Streptococcus oralis*, and *Veillonella dispar* (Table S4). Other species in *Actinomyces*, *Bifidobacterium*, *Campylobacter*, *Filifactor*, *Haemophilus*, *Lachnoanaerobaculum*, *Lactobacillus*, *Leptotrichia*, *Neisseria*, *Prevotella*, *Rothia*, *Selenomonas*, *Streptococcus*, *Tannerella*, *Veillonella*, or *Scardovia* did not coaggregate with *C. matruchotii* in this assay (Table S4).

### 3.6. C. matruchotii Coadhesion Partners in Surface-Based Assays

**Figure 4:**
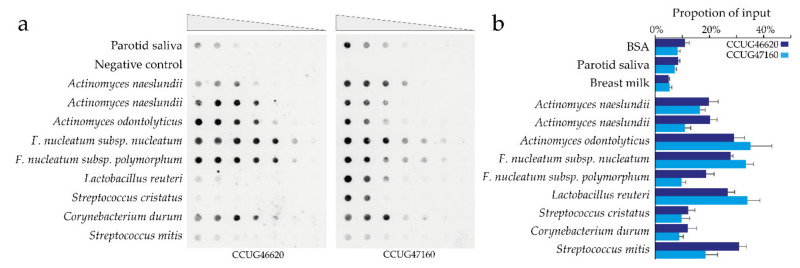
Surface-based *C. matruchotii* coadhesion partners. (**a**) Serial dilution of potential coadhesion partners for *C. matruchotii* (50 μL of OD_600_ from 0.5 to 0.004) were evaluated for binding of FITC-labeled *C. matruchotii*. The signal represents the binding of labeled *C. matruchotii* to filters coated with potential partner bacteria. (**b**) Proportion ^35^S-labeled *C. matruchotii* binding to saliva-coated or saliva-partner bacteria-coated hydroxyapatite beads. The bars represent mean and SD of three independent experiments.

Next, the same set of species were tested as potential coadhesion partners for *C. matruchotii* using an overlay assay. Coadhesion occurred for *A. naeslundii*, *A. odontolyticus*, *F. nucleatum* subsp. *nucleatum*, *F. nucleatum* subsp. *polymorphum*, *C. durum*, *L. reuteri*, *S. cristatus*, and *S. mitis* (Figure S1). A few more species (i.e., *A. actinomycetemcomitans*, *Rothia dentocariosa*) exhibited weak coadhesion, but the majority had limited signs of coadhesion (Figure S1). Serial dilution of the potential coadhesion partners *A. naeslundii*, *A. odontolyticus*, *F. nucleatum* subsp. *nucleatum*, *F. nucleatum* subsp. *polymorphum*, *L. reuteri*, *S. cristatus*, *C. durum*, and *S. mitis* exhibited interactions, though with some *C. matruchotii* strain variation for strains CCUG46620 and CCUG47160 (Figure 4a; Table S4).

### 3.7. Actinomyces spp. Recruitment of C. matruchotii to Hydroxyapatite

The next step was to examine whether *C. matruchotii* could utilize bacterial coadhesion partners to bind to tooth surfaces using a saliva and partner bacteria-coated hydroxyapatite bead assay. The test strains were selected from our surface overlay assay. *A. naeslundii*, *A. odontolyticus*, *F. nucleatum* subsp. *nucleatum, F. nucleatum* subsp. *polymorphum*, *L. reuteri*, and *S. mitis* exhibited a significant ability to recruit *C. matruchotii* to the saliva-coated hydroxyapatite bead. *C. durum* and *S. cristatus* species could not mediate *C. matruchotii* recruitment. The recruitment pattern was largely similar for the two tested *C. matruchotii* strains (CCUG46620 and CCUG47160) (Figure 4b; Table S4).

### 3.8. Carbohydrate Mediated Fluid Phase Coaggregation between C. matruchotii and Partner Bacteria

To understand the planktonic bacteria–bacteria interaction mode between *C. matruchotii* and *A. naeslundii*, *A. odontolyticus*, *F. nucleatum* subsp. *nucleatum*, *F. nucleatum* subsp. *polymorphum*, *F. peridonticum*, or *P. gingivalis*, heat treatment was used to denature surface proteins and differentiate between protein–protein or protein–carbohydrate interactions. Heat treatment of *C. matruchotii* disrupted the coaggregation with *A. naeslundii* and *A. odontolyticus* (Table S5), and heat treatment of *F. nucleatum* subsp. *nucleatum*, *F. nucleatum* subsp. *polymorphum*, *F. peridonticum*, or *P. gingivalis* abolished aggregation with *C. matruchotii.* This suggests that *C. matruchotii* may utilize a protein to bind a carbohydrate structure on *Actinomyces* spp. and a carbohydrate epitope to interact with a protein on *F. nucleatum* subsp. *nucleatum*, *F. nucleatum* subsp. *polymorphum, F. peridonticum*, and *P. gingivalis*.

### 3.9. Glycolipid Binding

To map potential carbohydrate ligands for *C. matruchotii* adhesion, a panel of monosaccharides (fructose, glucose, galactose) and disaccharides (sucrose, maltose, lactose) were screened for inhibition of coaggregation. None of the tested monosaccharides or disaccharides had a visual effect on the coaggregation between *C. matruchotii* and *A. naeslundii* or *A. odontolyticus* (data not shown).

**Figure 5:**
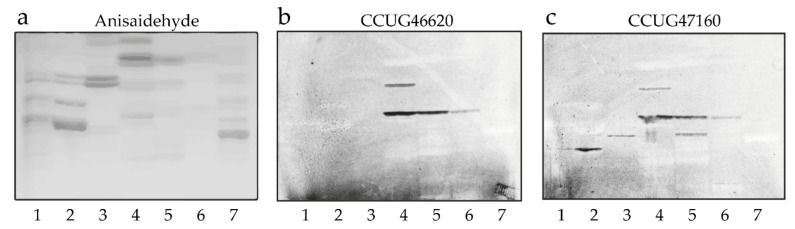
Screening for *C. matruchotii* carbohydrate recognition by binding to glycolipids on thin-layer chromatograms. (**a**) Chemical detection by anisaldehyde. (**b**) Autoradiograms obtained by binding of *C. matruchotii* strain CCUG46620 and (**c**) *C. matruchotii* strain CCUG47160, followed by autoradiography for 12 h. The solvent system used was chloroform/methanol/water (60:35:8, by volume). Lane 1, non-acid glycosphingolipids of human blood group AB erythrocytes, 40 μg; lane 2, non-acid glycosphingolipids of human blood group O erythrocytes, 40 μg; lane 3, non-acid glycosphingolipids of human granulocytes, 40 μg; lane 4, non-acid glycosphingolipids of rabbit intestine, 40 μg; lane 5, non-acid glycosphingolipids of human meconium, 40 μg; lane 6, non-acid glycosphingolipids of rat intestine, 40 μg; lane 7, non-acid glycosphingolipids of sheep intestine, 40 μg.

To further evaluate potential carbohydrate ligands for *C. matruchotii*, a number of glycolipid binding experiments were performed. In the first set of binding experiments, we used mixtures of glycolipids from different sources in order to expose the bacteria to a large number of variant carbohydrate sequences. We obtained selective binding to compounds that, on thin-layer chromatograms, migrated in the triaosylceramide to hexaosylceramide regions in the non-acid fractions from human blood group O erythrocytes, granulocytes, and meconium, as well as rabbit and rat intestines (Figure 5, lanes 2–6). There was also occasional binding to fast-migrating compounds in the total non-acid glycolipid fractions. However, this was judged to be a non-specific interaction caused by the large amounts of these compounds on the chromatograms because no binding to any monoglycosylceramides or diglycosylceramides was obtained when using defined amounts of pure glycolipids (see below). In addition, no binding was observed when binding of the two *C. matruchotii* strains to acid glycolipids was tested (data not shown).

**Figure 6:**
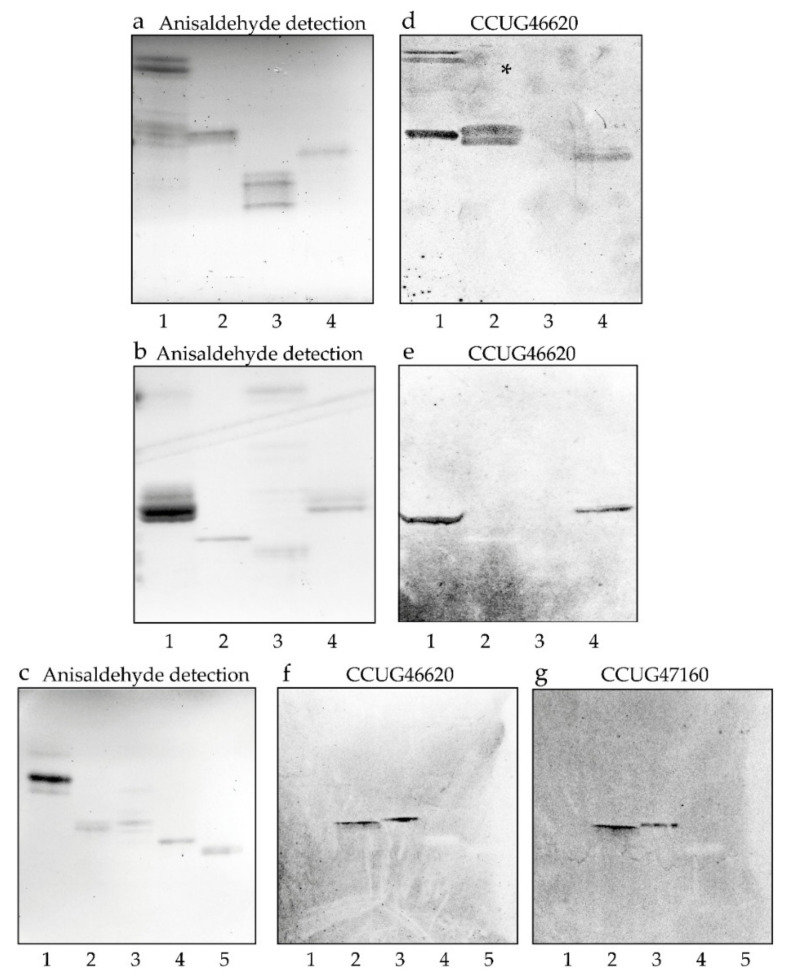
Binding of *C. matruchotii* to reference glycosphingolipids on thin-layer chromatograms. Chemical detection by anisaldehyde (**a**–**c**), and autoradiograms obtained by binding of *C. matruchotii* strain CCUG46620 (**d**–**f**) and *C. matruchotii* strain CCUG47160 (**g**), followed by autoradiography for 12 h. The solvent system used was chloroform/methanol/water (60:35:8, by volume). The lanes on (**a**,**d**) are: Lane 1, non-acid glycosphingolipids of rat intestine, 40 μg; lane 2, isoglobotriaosylceramide (Galα3Galβ4Glcβ1Cer), 4 μg; lane 3, Galili pentaosylceramide (Galα3Galβ4GlcNAcβ3Galβ4Glcβ1Cer) and Galili heptaosylceramide (Galα3Galβ4GlcNAcβ3Galβ4GlcNAcβ3Galβ4Glcβ1Cer), 4 μg; lane 4, Galα3-isoglobotriaosylceramide (Galα3Galα3Galβ4Glcβ1Cer), 4 μg. The lanes on (**b**,**e**) are: Lane 1, triglycosylceramides of human meconium (globotriaosylceramide, Galα4Galβ4Glcβ1Cer and lactotriaosylceramide, GlcNAcβ3Galβ4Glcβ1Cer, mixture), 10 μg; lane 2, H type 1 pentaosylceramide (Fucα2Galβ3GlcNAcβ3Galβ4Glcβ1Cer), 4 μg; lane 3, non-acid glycosphingolipids of porcine intestine, 20 μg; lane 4, lactotriaosylceramide (GlcNAcβ3Galβ4Glcβ1Cer), 4 μg. The lanes on (**c**,**f**,**g**) are: Lane 1, globotriaosylceramide (Galα4Galβ4Glcβ1Cer), 8 μg; lane 2, lactotetraosylceramide (Galβ3GlcNAcβ3Galβ4Glcβ1Cer), 4 μg; lane 3, neolactotetraosylceramide (Galβ4GlcNAcβ3Galβ4Glcβ1Cer), 4 μg; lane 4, H type 2 pentaosylceramide (Fucα2Galβ4GlcNAcβ3Galβ4Glcβ1Cer), 4 μg; lane 5, Le^x^ pentaosylceramide (Galβ4(Fucα3)GlcNAcβ3Galβ4Glcβ1Cer), 4 μg.

As the glycolipid mixtures have been characterized in previous studies [29,30,31,32,33], we examined the binding of *C. matruchotii* to a number of representative, structurally characterized reference compounds from our glycolipid library. The results from these binding assays are exemplified in Figure 6 and summarized in Table 2. Two triglycosylceramides, isoglobotriaosylceramide (Figure 6d, lane 2) and lactotriaosylceramide (Figure 6e, lane 4), were recognized by the bacteria (see Table 2 for glycolipid structures). In the case of isoglobotriaosylceramide, a substitution of the terminal Gal by Galα in the 3-position was tolerated with retained binding of the bacteria (Galα3-isoglobotriaosylceramide; Figure 6d, lane 4), whereas a substitution with GalNAcβ in the 3-position abrogated the binding (isoglobotetraosylceramide; Table 2, No. 9). No binding to other glycolipids with terminal Galα3 was observed, such as the Galili penta- and heptaosylceramides (Figure 6d, lane 3), demonstrating that a terminal Galα3 was not enough to support binding of the bacteria.

In the case of lactotriaosylceramide, *C. matruchotii* also bound when the terminal GlcNAc was substituted with Galβ in the 3-position, creating lactotetraosylceramide (Figure 6f,g, lane 2), or in the 4-position, creating neolactotetraosylceramide (Figure 6f,g, lane 3). Neolactohexaosylceramide (Table 2, No. 16) was also recognized by the bacteria. Further elongation of the terminal Gal of lactotetraosylceramide or neolactotetraosylceramide by Fucα in the 2-position (H type 1 pentaosylceramide, Figure 6e, lane 2; and the H type 2 pentaosylceramide, Figure 6f,g, lane 4) eliminated binding of *C. matruchotii*. Substitution of the GlcNAc of neolactotetraosylceramide with Fucα in the 3-position also blocked bacterial binding (Le^x^ pentaosylceramide; Figure 6f,g, lane 5).

## 4. Discussion

The present study surveyed the presence of *C. matruchotii* in saliva and tooth biofilm from birth to young adulthood and explored phenotypical characteristics of *C. matruchotii* related to its presence in saliva and establishment in tooth biofilms. The main findings are that the species establishes after 3 months of age and then reaches nearly 100% by early adulthood. *C. matruchotii* is enriched in tooth biofilms compared to saliva but does not bind directly to the tooth saliva pellicle and may need an adjuvant component to establish itself. *C. matruchotii* recognition of lactotriaosylceramide, lactotetraosylceramide, and neolactotetraosylceramide may mediate binding to human tissues.

Our findings also support that *C. matruchotii* is part of the core tooth microbiota in humans. Due to the relatively late colonization, establishment may be associated with tooth eruption, but it is not evidently associated with the number of teeth in early adulthood. Saliva binding in the planktonic phase, but with a limited ability to bind saliva-coated tooth surfaces, suggests binding to a non-tooth absorbing protein or glycoprotein within the saliva, or hidden epitopes after absorption to the tooth surface and that the oral biofilm establishment may build on partner bacteria-dependent recruitment. This would be in line with the prerequisite of tooth eruption and formation of a tooth biofilm before *C. matruchotii* establishment. Theoretically, binding may also be mediated by a structurally modified tooth pellicle component, but this was not tested in the present study.

*C. matruchotii* has been indicated to be in close proximity with *Actinomyces* spp. at the tooth surface [3]. In vivo, *A. naeslundii* was associated with the abundance of *C. matruchotii* at all ages and, in vitro, *A. naeslundii* bound *C. matruchotii* in both planktonic and overlay assays and possessed the ability to recruit *C. matruchotii* to the tooth surface. We can hypothesize that *A. naeslundii*, which is an initial tooth colonizer [34], binds to the saliva pellicle [35] and forms a layer that provides *C. matruchotii* attachment sites. In addition to *A. naeslundii*, *C. matruchotii* associated with *F. nucleatum* subsp. *polymorphum, Leptotrichia* sp. *HMT 392*, and *Tannerella* sp. *HMT 286* in in vivo and in vitro binding assays suggests that *Fusobacterium*, *Porphyromonas*, *Aggregatibacter,* and *Streptococcus* may directly interact with *C. matruchotii*. To some extent, this is in line with in vivo localization in which the neighbors along the *C. matruchotii* filament consisted of *Fusobacterium*, *Leptotrichia*, and *Capnocytophaga,* and at its distal end *Streptococcus* and *Porphyromonas*, with frequently observed *Haemophilus*-*Aggregatibacter* and *Neisseriaceae* [3]. Notably, *Capnocytophaga* spp. were associated with the in vivo abundance of *C. matruchotii* at all ages but did not directly bind to *C. matruchotii*. The binding properties of *C. matruchotii* suggest both protein and carbohydrate interactions with its bacterial partners. Similar dual mechanisms have been observed for *A. naeslundii* [36], but further studies are required to dissect the *C. matruchotii* bacterial interaction network.

Glycolipid binding assays demonstrated selective binding of *C. matruchotii* to isoglobotriaosylceramide, Galα3-isoglobotriaosylceramide, lactotriaosylceramide, lactotetraosylceramide, and neolactotetraosylceramide. Isoglobotriaosylceramide is not likely to be a physiologically relevant ligand for *C. matruchotii* in humans because this glycolipid has not been found in human tissues [37]. Galα3-isoglobotriaosylceramide has been characterized in cat intestine [38], but not yet in humans. *C. matruchotii* ligands in humans are most likely lactotriaosylceramide, lactotetraosylceramide, and neolactotetraosylceramide. Lactotetraosylceramide has been identified in the human gastrointestinal tract [39,40,41], whereas neolactotetraosylceramide is widely distributed in human tissues and has been characterized in erythrocytes [42], neutrophils [33], brain [43], and stomach [40]. Lactotriaosylceramide is the precursor of both lactotetraosylceramide and neolactotetraosylceramide and should also be present in these tissues. Furthermore, the terminal Galβ4GlcNAcβ sequence of neolactotetraosylceramide is a common core structure in the carbohydrate chains of glycoproteins [44].

The current study has some strengths and limitations that should be recognized and considered when evaluating the results. The strengths include a combination of in vivo clinical data and in vitro experimental data to support the conclusions and the wide age range from earliest infancy to early adulthood. In addition, two different *C. matruchotii* strains were used to cover some within-species genetic variations. The two strains mainly presented overlapping results, but also some phenotypic variation. In addition, for the majority of tested *C. matruchotii* bacterial binding partners, a limited number of strains within in each species were evaluated, limiting the coverage of intra-species variations.

The limitations include a relatively low number of participants with milk and feces samples; however, as the results were consistent among the samples analyzed, these samples rendered conclusive results on the low detection prevalence and abundance. In addition, the use of next generation sequences (NGS) for the identification of microorganisms is potentially associated with errors at various steps, including PCR amplification, sequencing, and bioinformatics. Furthermore, NGS identification includes dead bacteria, and, finally, self-reported information is prone to bias [45].

*C. matruchotii* colonizes the oral cavity, the skin [46], and likely the nose and nasopharynx, but is not detected in the gastrointestinal tract or human milk, but the association between *C. matruchotii* and health is still unclear. Interestingly, abundance of the *Corynebacterium* genus was negatively associated with head and neck squamous cell cancer risk, which also parallels less smoking [47]. We also found smoking to be associated with reduced levels of *C. matruchotii,* which also coincides with some previous studies [12,15], but not others [17,48]. Notably, *C. matruchotii* has been suggested to be a low immunogenic species compared to other oral bacteria [49], and the finding that *C. matruchotii* possesses the ability to bind glycolipids isolated from tissues is of interest for future studies. Genetic tools are required to further understand *C. matruchotii* [12,50]. Such tools were valuable when the *C. matruchotii* MdbA gene was characterized, indicating that *C. matruchotii* possesses the ability to catalyze disulfide bond formation in vitro, and has a similar function as the *Corynebacterium diphtheriae* MdbA gene, which is required to maintain normal cell growth and morphology, toxin production, and pilus assembly [12].

In conclusion, the present study shows that *C. matruchotii* establishes after 3 months of age and has reached nearly 100% at early adulthood. *C. matruchotii* oral biofilm establishment is likely to require both tooth eruption and formation of a pre-existing bacterial biofilm, potentially by *A. naeslundii*. In addition, *C. matruchotii* has the ability to interact with glycolipids present on human cells and exhibits specific binding to lactotriaosylceramide, lactotetraosylceramide, neolactotetraosylceramide, isoglobotriaosylceramide, and Galα3-isoglobotriaosylceramide.

## Figures and Tables

**Table 1:** *Corynebacterium matruchotii* detection in saliva, breast milk, and feces by age, and tooth biofilm samples at 18 years of age.

	Age at Sampling					
	2 Days	3 Months	18 Months	3 Years	5 Years	18–23 Years
**Saliva**						
Numbers (% female)	206 (47)	155 (46)	132 (42)	140 (45)	116 (49)	175 (51)
Smoker, number						8
Prevalence carrier, %	1.9	0	50	61.4	67.2	98.3
Relative abundance, median; mean	3.0; 4.1	0.0; 0.0	0.26; 0.46	0.19; 0.34	0.08; 0.20	0.32; 0.54
**Tooth Biofilm**						
Numbers (% female)						45 (60)
Smoker, number						4
Prevalence carrier, %						100
Relative abundance, median; mean						5.2; 6.5
**Breast Milk**						
Numbers (% female)		113 (46)				
Prevalence carrier, %		10.1 (12)				
Relative abundance, median; mean		0.10; 0.10				
**Feces**						
Numbers (% female)		135 (61)			52 (48)	
Prevalence carrier, %		0			0	
Relative abundance, median; mean		0			0	

Relative abundances are given as median; mean. Relative abundance of *C. matruchotii* is given as mean values (i.e., % of all reads in the participants in whom *C. matruchotii* was present).

**Table 2:** Binding of *C. matruchotii* CCUG46620 and CCUG47160 to glycolipids on thin-layer chromatograms.

No	Trivial Name	Structure	Binding ^a^
1.	Galactosylceramide	Galβ1Cer	-
2.	Sulfatide	SO_3_-Galβ1Cer	-
3.	LacCer	Galβ4Glcβ1Cer	-
4.	Neu5Gc-GM3	Neu5Gcα3Galβ4Glcβ1Cer	-
5.	Globotri	Galα4Galβ4Glcβ1Cer	-
6.	Isoglobotri	Galα3Galβ4Glcβ1Cer	+
7.	Galα3-isoglobotri	Galα3Galα3Galβ4Glcβ1Cer	+
8.	Globotetra	GalNAcβ3Galα4Galβ4Glcβ1Cer	-
9.	Isoglobotetra	GalNAcβ3Galα3Galβ4Glcβ1Cer	-
10.	Galili penta	Galα3Galβ4GlcNAcβ3Galβ4Glcβ1Cer	-
11.	Galili hepta	Galα3Galβ4GlcNAcβ3Galβ4GlcNAcβ3Galβ4Glcβ1Cer	-
12.	Forssman	GalNAcα3GalNAcβ3Galα4Galβ4Glcβ1Cer	-
13.	Lactotri	GlcNAcβ3Galβ4Glcβ1Cer	+
14.	Lactotetra	Galβ3GlcNAcβ3Galβ4Glcβ1Cer	+
15.	Neolactotetra	Galβ4GlcNAcβ3Galβ4Glcβ1Cer	+
16.	Neolactohexa	Galβ4GlcNAcβ3Galβ4GlcNAcβ3Galβ4Glcβ1Cer	+
17.	H type 1 penta	Fucα2Galβ3GlcNAcβ3Galβ4Glcβ1Cer	-
18.	H type 2 penta	Fucα2Galβ4GlcNAcβ3Galβ4Glcβ1Cer	-
19.	Le^x^ penta	Galβ4(Fucα3)GlcNAcβ3Galβ4Glcβ1Cer	-
20.	A type 1 hexa	GalNAcα3(Fucα2)Galβ3GlcNAcβ3Galβ4Glcβ1Cer	-
21.	Le^b^ hexa	Fucα2Galβ3(Fucα4)GlcNAcβ3Galβ4Glcβ1Cer	-

^a^ + denotes a binding when 4 μg of the glycolipid was applied on the thin-layer chromatogram, whereas - denotes no binding, even at 4 mg.

## Supplementary Materials

The following are available online at https://www.mdpi.com/2076-2607/8/11/1780/s1. Figure S1: Surface-based C. matruchotii coadhesion partners, Table S1: List of bacterial species and strains with culture medium and conditions for use in aggregation and adhesion experiments, Table S2: C. matruchotii in saliva by various lifestyle factors, Table S3: Candidate partner bacteria for C. matruchotii in oral biofilm formation, Table S4: Ability of bacterial partners to coaggregate, coadhere, and recruit C. matruchotii to the tooth surface, Table S5: Bacterial–bacterial binding properties.

## Appendix A

Genomic DNA was extracted from saliva, saliva swabs, tooth biofilm, milk, and feces samples, and positive controls created using the GenElute™ Bacterial Genomic DNA Kit (Sigma–Aldrich, St. Louis, MO, USA) with the addition of lysozyme and mutanolysin, RNase, and proteinase K to ensure harvest from both Gram-positive and Gram-negative bacteria. Bacterial 16S rRNA gene amplicons were generated from the v3–v4 (357F-forward TACGGGAGGCAGCAG and 800R-reverse CCAGGGTATCTAATCC primers) variable regions of the 16S rRNA gene, sequenced on an Illumina MiSeq platform, and demultiplexed using deML [51]. Amplicon primers were removed from the obtained sequences and quality checked in QIIME 2 (qiime2.org) [52]. Length trimming, denoising, and chimera and PhiX removal were achieved using DADA2 [53] within QIIME2, resulting in identification of amplicon sequence variants (ASVs) per sample [54]. Default parameters in DADA2 were used with left trimming of 13 bp for both forward and reversed reads, and right trimming at 230 bp for the reversed sequences and 268 bp for the forward sequences. ASVs with only one read were filtered, and species or phylotypes represented by at least 25 reads with 98.5% identity in the extended Human Oral Microbiome Database (eHOMD, http://www.homd.org) [55,56] were retained. For simplicity, both named and unnamed phylotypes are referred to as species in the text. Mixtures of known mock bacteria were used as positive controls and sterile water as the negative control.
