# Supplementary figures and images for: Corynebacterium matruchotii Demography and Adhesion Determinants in the Oral Cavity of Healthy Individuals
Source: Microorganisms. 2020 Nov 13;8(11):1780. doi: 10.3390/microorganisms8111780 (PMC7697164; doi:10.3390/microorganisms8111780)

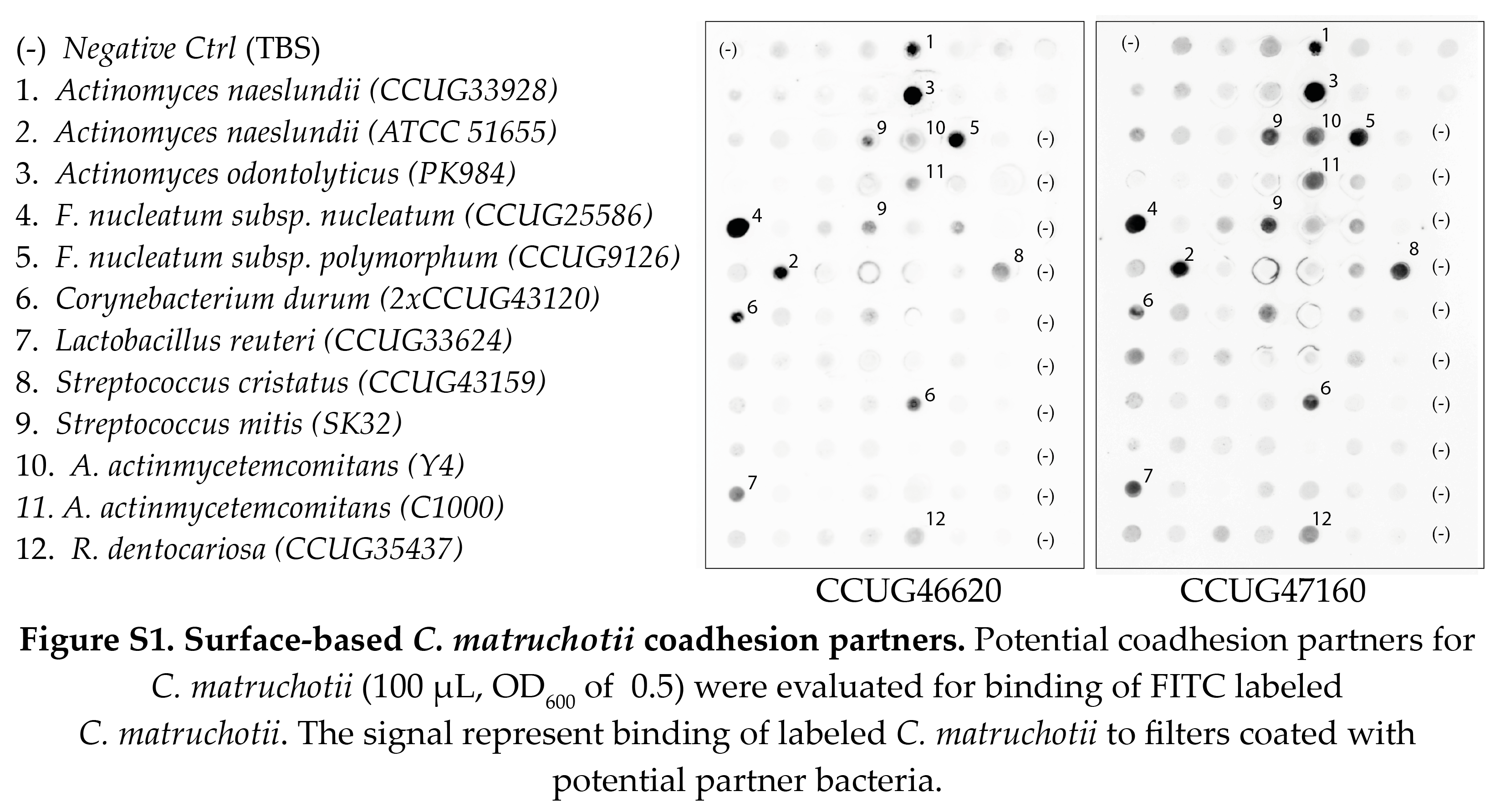

Supplement: Supplementary file 1 [file microorganisms-08-01780-s001.zip › Microorganis_Figure S1.tif]
